# The spectrum of *C9orf72* repeat lengths in Portuguese frontotemporal dementia and amyotrophic lateral sclerosis patients: from pathogenic expansions to intermediate alleles

**DOI:** 10.3389/fnins.2026.1913909

**Published:** 2026-08-31

**Authors:** Maria Rosário Almeida, Miguel Tábuas-Pereira, Anabela Matos, Ana Santos, Carolina Teixeira, Inês Baldeiras, Marisa Lima, Isabel Santana

**Affiliations:** 1 CIBB – Center for Innovative Biomedicine and Biotechnology, University of Coimbra, Coimbra, Portugal; 2 CNC-UC – Center for Neuroscience and Cell Biology, University of Coimbra, Coimbra, Portugal; 3 Department of Neurology, Hospital Universitário de Coimbra, Unidade Local de Saúde de Coimbra (ULS-Coimbra), Coimbra, Portugal; 4 Faculty of Medicine, University of Coimbra, Coimbra, Portugal

**Keywords:** ALS, *C9orf72* expansion, FTD, FTD-ALS, intermediate alleles

## Abstract

**Introduction:**

Frontotemporal dementia and amyotrophic lateral sclerosis are clinically distinct disorders that share a common genetic etiology, with pathogenic hexanucleotide repeat expansions in the *C9orf72* gene representing the most frequent genetic cause of both conditions. In this study we aim to determine the frequency of *C9orf72* repeat expansions in patients with FTD and/or ALS and their at-risk relatives from central Portugal, and to analyze the distribution and repeat size of intermediate alleles within this cohort investigating their potential pathogenicity.

**Methods:**

Between January 2016 and January 2026, *C9orf72* repeat expansions were assessed in 610 patients with the clinical presentation within the spectrum of FTD/ALS and in 63 asymptomatic relatives. *C9orf72* repeat expansions were identified using a two-step PCR protocol comprising STR-PCR and RP-PCR, followed by repeat-length sizing with a commercial kit. Capillary electrophoresis traces were subsequently analyzed using AmplideX® PCR/CE Reporter software. Furthermore, carriers of intermediate alleles underwent comprehensive clinical, fluid-biomarker, and neuropsychological characterization to investigate the potential pathogenicity of these alleles.

**Results:**

Pathogenic *C9orf72* expansions were identified in 11% of patients. The vast majority carried long expansions (>145 repeats), whereas three patients exhibited shorter expansions ranging from 38 to 78 repeats. Intermediate alleles were found in seven subjects with heterogeneous clinical presentations. Clinically, these individuals were indistinguishable from carriers of longer expansions.

**Discussion:**

*C9orf72* hexanucleotide repeat expansions are a major genetic cause of FTD, ALS, and FTD–ALS in this cohort of patients, highlighting the importance of genetic testing for accurate counselling and clinical management. These findings support routine *C9orf72* testing in all patients with these disorders, particularly those of European ancestry. Our findings also support the emerging view that intermediate C9orf72 repeat expansions may act as a genetic risk factors contributing to a broad spectrum of clinical phenotypes.

## 1 Introduction

Frontotemporal dementia (FTD) and Amyotrophic lateral sclerosis (ALS) are two clinically distinct entities. In FTD, neurodegeneration affects the frontal and temporal lobes resulting in changes in behavior, social awareness, and language (The Lund and Manchester Groups, 1994). FTD is the second most common cause of early onset dementia with a prevalence of 15 cases per 100,000 individuals aged 45–65 years (Knopman and Roberts, 2011). Although most FTD cases occur sporadically, approximately 30–40% of patients have a family history of the disease (Seelaar et al., 2008). FTD patients can be classified into three main clinical subtypes according to their symptomatology: behavioral variant frontotemporal dementia (bvFTD), in which changes in behavior and social conduct predominate, including loss of social awareness and poor impulse control; and two language variants, semantic dementia (SD) (Snowden et al., 1989; Hodges and Patterson, 2007) and progressive non-fluent aphasia (PNFA) (Mesulam, 1982; Mesulam, 2001; Gorno-Tempini et al., 2004). Amyotrophic lateral sclerosis (ALS) is a neurodegenerative disorder that affects both upper and lower motor neurons leading to hyperreflexia, spasticity, fasciculations, progressive weakness and muscular atrophy (van Es et al., 2017; Hardiman et al., 2017). Additionally, a significant number of patients develop cognitive and/or behavioral impairment due to prefrontal involvement (Phukan et al., 2012). Approximately 10% of all ALS cases are familial, while the remaining 90% are sporadic (Ferrari et al., 2011). It is the most common adult-onset motor neuron disease, with a prevalence of around five per 100,000 individuals with a typical onset after the age of 60 (Worms, 2001; Horton et al., 2014). Based on the clinical, genetic, and neuropathological overlap between these two diseases, ALS and FTD are now recognized as two extremes of the FTD/ALS spectrum (Van Langenhove et al., 2012). Indeed, both disorders can be present within the same family or even within the same individual. Notably, approximately 30% of FTD patients develop motor dysfunction, and up to 50% of patients with ALS exhibit symptoms associated with FTD (Ng et al., 2015). A mutation in chromosome 9 open reading frame 72 gene (*C9orf72*) has been identified as a common genetic cause of both ALS and FTD. The link of ALS and FTD to chromosome 9 was first reported in 2006 in two independent studies (Vance et al., 2006; Morita et al., 2006). Subsequently, linkage to the same chromosome 9p region was found in additional independent FTD-ALS kindreds, defining a shared candidate region of 7.7 Mb containing 52 candidate genes (Momeni et al., 2006; Valdmanis et al., 2007; Luty et al., 2008; Le Ber et al., 2009). In 2011, Boxer et al. described a new FTD-ALS family definitively linked to chromosome 9p with novel clinical, neuroimaging and neuropathological findings, whose disease-associated haplotype reduces the size of the candidate region linked to FTD-ALS to a 3.7 Mb interval on chromosome 9p containing only 10 genes (Boxer et al., 2011). Soon afterwards, it was identified the cause of the chromosome 9 linked FTD and ALS to be an expansion of a GGGGCC (G4C2) hexanucleotide repeat in a noncoding region of the *C9orf72* (DeJesus-Hernandez et al., 2011; Renton et al., 2011; Gijselinck et al., 2012). Since then, multiple studies have confirmed this association, establishing the *C9orf72* repeat expansion as the most common genetic cause of ALS in European populations, accounting for 39.6% of familial and 4.6% of sporadic cases (Zou et al., 2017). It is also a major cause of FTD, occurring in 18.5% of familial and 6.3% of sporadic FTD cases (van der Zee et al., 2013), and has been reported in up to 88% of familial ALS–FTD cases (Majounie et al., 2012; Cruts et al., 2013). However, geographic variability in the frequency of *C9orf72* repeat expansions has been observed worldwide even across European countries and regions. The highest frequencies have been reported in Scandinavian countries (Smith et al., 2013; Lindquist et al., 2013), whereas the expansion is generally rare in Asian populations (Tsai et al., 2012; Konno et al., 2013; Jang et al., 2013; Zou et al., 2013). An individual carrying a pathological expansion can develop either FTD, ALS, or both with variable clinical expression (Roggenbuck and Fong, 2020). Irrespective of the presenting symptoms, the onset age of *C9orf72* repeat expansion carriers was variable but commonly falls in the 50s to early 60s for both conditions (Gossye et al., 2015). Importantly, multiple studies have demonstrated that *C9orf72* repeat expansion shows age-dependent and incomplete penetrance, as clinical manifestations are rare before age 35, increase progressively with advancing age, reach approximately 50% penetrance by 58 years, and rise to roughly 90–100% by age 80 (Benussi et al., 2015; Murphy et al., 2017; Crook et al., 2019). Survival time is variable and mainly associated with an individual’s clinical features. For ALS, *C9orf72* repeat expansions were associated with an average disease duration of 2.9 years (Cammack et al., 2019). In *C9orf72*-FTD patients, disease duration averages 6.4 years (Moore et al., 2020). However, when ALS symptoms emerge in individuals with FTD, survival is markedly reduced to on average 1.8 years (Van Mossevelde et al., 2018). Some investigators have reported an inverse correlation between age at disease onset and successive generations in *C9orf72* families, suggesting genetic anticipation, with younger generations exhibiting earlier onset (Van Mossevelde et al., 2017), while others have failed to replicate this finding (Gijselinck et al., 2016). Therefore, no robust conclusions can currently be drawn, as the evidence remains contradictory. This discrepancy may reflect two key challenges. First, accurately measuring the extremely large *C9orf72* repeat expansions across generations is technically difficult, limiting evidence for genetic anticipation. Second, intergenerational differences in repeat length and associated methylation levels within the *C9orf72* promoter region may also contribute to the observed variability. Most healthy individuals carry alleles between 2 and 20 repeats in length, whereas patients with *C9orf72*-associated ALS and FTD typically harbor expansions ranging from hundreds to several thousand G4C2 units (DeJesus-Hernandez et al., 2011; Renton et al., 2011; Gijselinck et al., 2012; Babić Leko et al., 2019). A small subset of patients carries shorter pathogenic expansions of 45–80 repeats, highlighting an apparent gap between these alleles and the much larger common expansions (Gijselinck et al., 2016). Although the precise cutoff distinguishing normal from pathogenic repeat alleles has not been definitively established, pathological threshold of 30 repeat units is currently proposed (Renton et al., 2011). Alleles in the intermediate range of 20–30 repeats potentially acting as risk alleles for disease and hence named intermediate alleles, but their pathogenic significance remains uncertain. However, there are published reports of familial segregation with disease of repeats in the intermediate size range between 20 and 29 repeats (van der Zee et al., 2013; Gómez-Tortosa et al., 2013). Indeed, intermediate alleles of 21–29 repeats have been reported in patients with ALS (García-Redondo et al., 2013; Iacoangeli et al., 2019), who show clinical phenotypes similar to those with >30 repeats (Byrne et al., 2014; Chen et al., 2016), as well as in FTD (Gómez-Tortosa et al., 2013), Parkinson disease (Nuytemans et al., 2013), and essential tremor (Jiao et al., 2013). These findings suggest that intermediate-sized repeats may represent a potential risk factor for a broad spectrum of neurodegenerative diseases. Hence, in this study we present the outcomes of the screening for *C9orf72* repeat expansion in patients with FTD and/or ALS and their at-risk family members from the central region of Portugal. Additionally, given the ongoing uncertainty regarding the pathogenicity of intermediate *C9orf72* repeat alleles, we analyzed the distribution and repeat size of intermediate alleles within our patient cohort. Carriers of intermediate alleles underwent comprehensive phenotypic evaluation, including clinical assessment, fluid biomarker analysis, and neuropsychological evaluation, to investigate the potential pathogenicity of these alleles.

## 2 Materials and methods

### 2.1 Participants

During the period between January 2016 and January 2026, a total of 673 subjects was investigated for the presence of *C9orf72* expansion at the Neurogenetics Laboratory of the Center for Neuroscience and Cell Biology (CNC) at the University of Coimbra. Of these, 610 subjects had a clinical presentation within the spectrum of FTD/ALS and 63 were asymptomatic family members who provided their written informed consent for being tested. Patients were referred from the Dementia and Neuromuscular Units of the Department of Neurology at Unidade Local de Saúde de Coimbra (ULS-Coimbra). All of the unaffected family members were referred from the Genetic Counseling Unit at the Medical Genetics Department of ULS-Coimbra.

### 2.2 Clinical evaluation

The diagnosis of FTD was based on the Lund and Manchester clinical criteria (The Lund and Manchester Groups, 1994; Neary et al., 1998), which were revised by the Work Group on Frontotemporal Dementia and Pick’s Disease (McKhann et al., 2001), and more recently according to the International Behavioral Variant Frontotemporal Dementia Criteria Consortium for behavioral variant FTD (bvFTD) (Rascovsky et al., 2011) and the proposed criteria for primary progressive aphasia (Gorno-Tempini et al., 2011). The ALS diagnosis was based on the revised El Escorial criteria (Brooks et al., 2000) and the Awaji recommendations (Costa et al., 2012), which emphasize incorporating electrodiagnostic findings to increase diagnostic sensitivity compared to the El Escorial criteria alone. More recently, diagnostic classification followed the Gold Coast criteria, which prioritize progressive motor impairment, evidence of upper and lower motor neuron dysfunction, and exclusion of alternative causes (Shefner et al., 2020).

### 2.3 Neuropsychological evaluation

The study participants underwent, whenever possible and depending on disease stage, a neuropsychological assessment protocol comprising: Mini Mental State Examination (MMSE) (Freitas et al., 2015), Montreal Cognitive Assessment (MoCA) (Freitas et al., 2011), and a comprehensive neuropsychological battery with normative data for the Portuguese population, the Battery of Lisbon for the Assessment of Dementia (BLAD) (Guerreiro, 1998). This battery includes some tests of the Wechsler Memory Scale (WMS) (Wechsler, 1945) and encompasses the following cognitive abilities: attention (Cancellation Task); verbal initiative (Semantic Fluency), motor and graphomotor initiatives; verbal comprehension (a modified version of the Token Test); verbal and non-verbal reasoning (Interpretation of Proverbs and the Raven’s Coloured Progressive Matrices – Ab series); orientation (spatial, temporal and social orientation); Visuoconstructive abilities (cube copy); basic written calculus; immediate memory (Digit Span Forward); visual memory (WMS Visual Reproduction Test); working memory (Digit Span Backward); learning and verbal memory (WMS Verbal Paired-Associate Learning, Logical Memory and Word Recall). All tests were administered and scored according to standardized procedures. Individual test scores were converted to z-scores. Cognitive impairment was defined as a z-score < −1.

### 2.4 *C9orf72* expansion analysis

DNA was isolated from peripheral blood using the NZY tissue gDNA Isolation kit (Nzytech, Portugal), as described by the manufacturer. The detection of the *C9orf72* expansion was performed using a 2-step PCR-based protocol as previously described (van der Zee et al., 2013). Initially, an STR-PCR assay was performed to identify the *C9orf72* alleles present. Samples showing only a single allele peak, were analyzed using in-house repeat-primed PCR (RP-PCR) to confirm the presence of an expanded allele. Repeat-length quantification was performed using the AmplideX® PCR/CE *C9orf72* kit (Asuragen, Inc.) for all cases in which an expansion was detected and for samples suspected to have an intermediate allele. This assay amplifies the G4C2 repeat region through a three-primer repeat-primed PCR configuration, enabling accurate sizing of normal, intermediate, and expanded alleles according to the manufacturer’s protocol. After denaturation, the amplicons were separated by capillary electrophoresis using a POP-7 polymer on an eight-capillary 3,500 Genetic Analyzer (Applied Biosystems™, Thermo Fisher Scientific, Waltham, MA, USA). The fragment sizes were determined using a ROX-1000 size standard and GeneMapper software, version 3.7 (Applied Biosystems™, Thermo Fisher Scientific, Waltham, MA, USA). The size of the PCR products in base pairs was converted to the corresponding number of G4C2 repeats using size and mobility conversion factors. A PCR control consisting of a mixture of four normal *C9orf72* alleles with known repeat lengths (two, five, eight, and ten G4C2 repeats) was used to create a linear calibration curve and determine correction factors for converting size (in base pairs) to the number of G4C2 repeats for each allele. In addition, the interpretation of the produced capillary electrophoresis (CE) trace files (FSA files) was also performed using AmplideX® PCR/CE Reporter software (Asuragen, Inc). We classified *C9orf72* alleles into three categories according to repeat size: normal (<20 repeats), intermediate (≥20–30 repeats), and pathogenic expansions (>30 repeats).

### 2.5 Fluid-biomarkers evaluation

CSF samples were collected as part of the patients’ routine clinical diagnostic examination. Standardized pre-analytical and analytical procedures were followed according to BIOMARKAPD guidelines for CSF-AD biomarkers (del Campo et al., 2012). Specifically, CSF samples were collected in sterile polypropylene tubes, immediately centrifuged at 1800 g for 10 min at 4 °C, aliquoted into 2 mL polypropylene tubes, and stored at −80 °C until analysis. CSF-AD biomarkers Aβ42, Aβ40, t-tau, and p-tau181 were measured automatically by commercially available immunoassays in the LUMIPULSE G600II platform (Fujirebio, Japan). External quality control of the assays was performed under the scope of the Alzheimer’s Association Quality Control Program for CSF Biomarkers (Mattsson et al., 2011). To classify CSF data, we applied the AT(N) scheme (Jack et al., 2018), which includes the Aβ42/40 ratio for assessing amyloid deposition (A), p-tau181 for evidence of Tau aggregation (T), and t-tau for neurodegeneration (N). Biomarker values were dichotomized as abnormal (+) or normal (−) using validated laboratory-specific cutoffs for the LUMIPULSE G600II platform (Leitão et al., 2019). CSF NfL determinations were done using a different aliquot than the one used for CSF-AD biomarkers. CSF was diluted 1:1 and NfL was quantified in duplicate using a validated ELISA assay (NF-light; Uman Diagnostics, Sweden), as previously reported (Silva-Spínola et al., 2022). When needed, samples were further diluted 1:10 and re-assayed. CSF NfL levels were considered normal/high according to the manufacturer’s age-adjusted cut-off values.

## 3 Results

**Figure 1:**
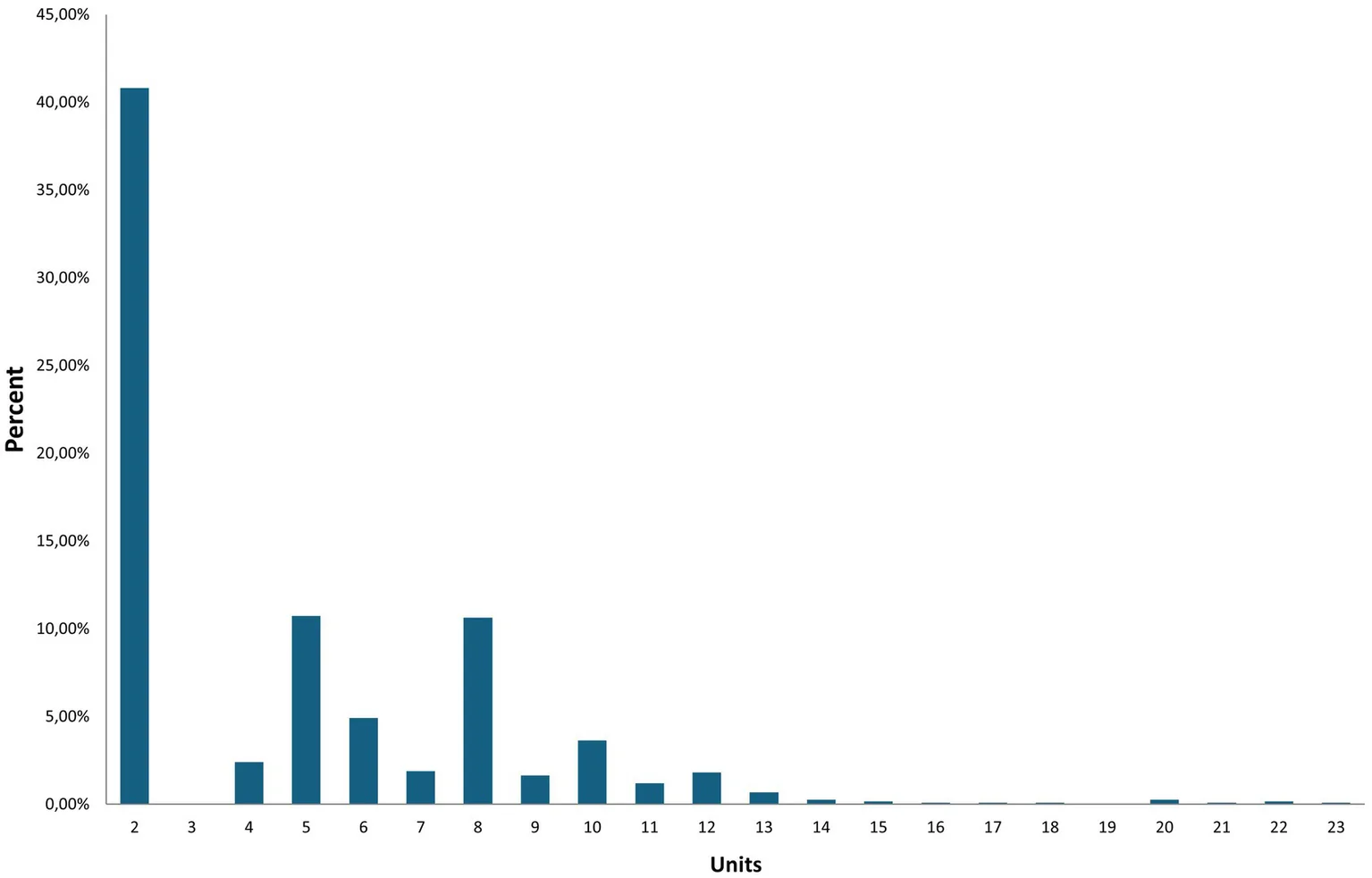
Distribution of *C9orf72* repeat-length alleles in our cohort.

**Figure 2:**
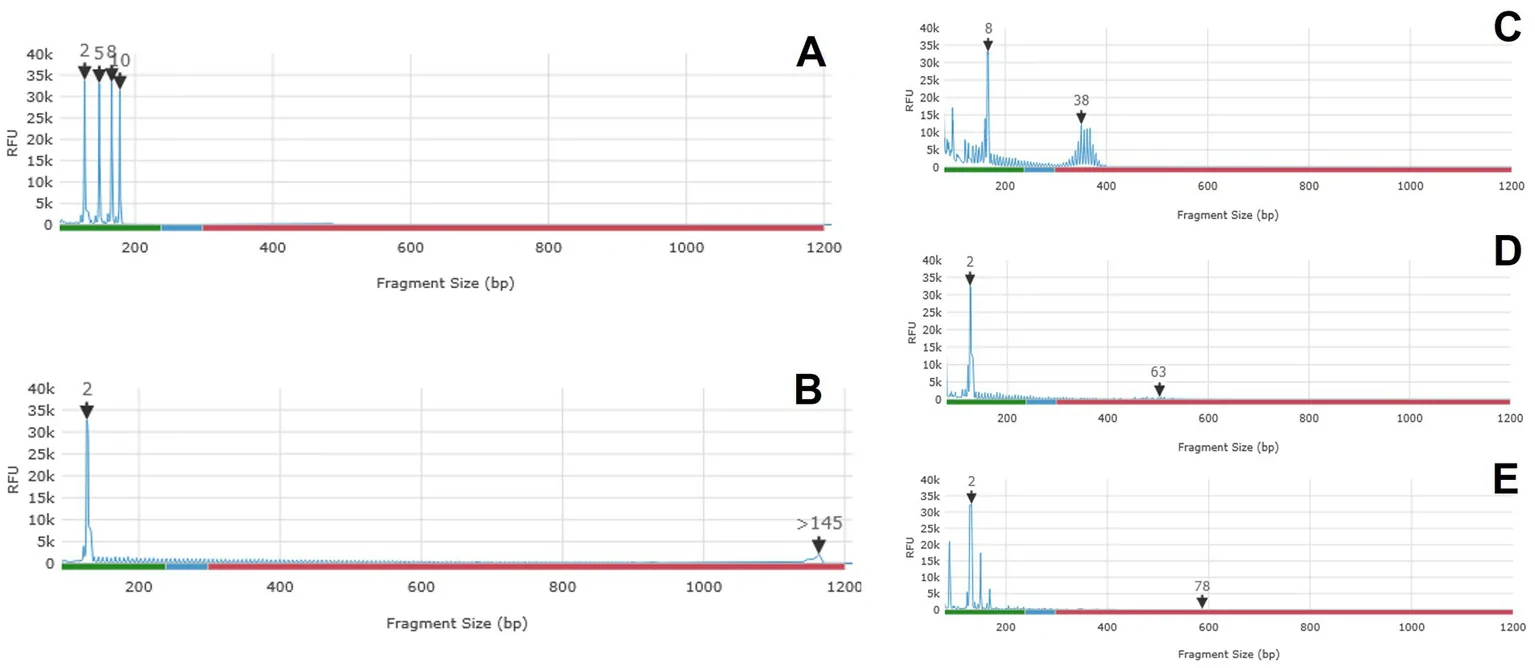
AmplideX® PCR/CE *C9orf72* Kit traces. **(A)** Kit control corresponding to 2, 5, 8, and 10 repeats; **(B)** Full-length *C9orf72* repeat expansion (>145 repeats); **(C–E)** Short *C9orf72* repeat expansion (38, 63, and 78 repeats, respectively).

We determined the frequency of the *C9orf72* hexanucleotide repeat expansions in a population of 610 patients within the spectrum of FTD/ALS and 63 asymptomatic family members. Demographic characteristics, clinical phenotypes, and *C9orf72* status for all individuals are summarized in Table 1. Female prevalence was higher in asymptomatic family members (60%) than in patients (44%). As expected, the patient group was significantly older than the asymptomatic group (*p* < 0.001). The patients’ clinical phenotype included 373 patients presented with FTD, 112 with ALS, 34 with combined FTD-ALS, 38 FTD-atypical parkinsonian features, and 53 with AD. The pathogenic *C9orf72* expansion was identified in 87 subjects, including 65 patients and 24 asymptomatic relatives, corresponding to 11 and 38% of the patient and family-member cohorts, respectively. The vast majority of both symptomatic and asymptomatic *C9orf72* repeat expansion carriers harbored large expansions (>145 repeats). Among asymptomatic relatives, one individual carried an allele with 20 repeat units, whereas among symptomatic individuals, only three exhibited smaller expansions ranging from 38 to 78 repeats. Although the vast majority of *C9orf72* repeat expansion carriers had long expansions (>145 repeats), three individuals exhibited smaller expansion sizes ranging from 38 to 78 repeats. Clinical phenotypes of *C9orf72* expansion carriers included 42 FTD patients (11% of all FTD cases), 16 ALS (14% of all ALS cases), and 7 FTD-ALS (21% of all FTD-ALS cases). However, *C9orf72* expansion was not detected in any patient with atypical parkinsonian features or Alzheimer’s disease (AD). Analysis of the overall allele size distribution in the cohort revealed that the 2-repeat allele was the most common, followed by the 5 and 8 repeat alleles, which together accounted more than 60% of all observed alleles (Figure 1). Alleles with 14 to 18 repeats were uncommon, representing about 0.7% of the total. The 3-repeat and 19-repeat alleles were not found in any patient. Regarding the expanded alleles, the methodology used does not allow quantitative sizing of alleles larger than 145 repeats. Accordingly, the vast majority of expanded alleles were classified as >145 repeats, although three shorter expanded alleles containing 38, 63, and 78 repeats were identified (Figure 2). Beyond the expanded alleles described above, we identified seven cases (6 patients and one asymptomatic) carrying an intermediate allele, typically defined as containing 20–30 repeats, with heterogeneous clinical presentations (Table 2). Cases #1 and #5 presented with FTD-ALS, with normal CSF-AD biomarkers and elevated NfL consistent with the patients’ phenotypes. No cognitive assessment was performed for Case #1 because of the advanced disease stage at the time of evaluation. For Case #5, only the MMSE was administered, yielding a score of 11, consistent with moderate dementia. Cases #2 and #7 presented with a phenotype consistent with primary progressive aphasia (PPA) and the language variant of Alzheimer’s disease, both patients also exhibiting positive cerebrospinal fluid biomarkers for AD indicating amyloid and tau pathology. Neuropsychological assessment showed similar phenotypes in both cases, with predominant naming and repetition deficits and associated memory impairment. However, disease stage differed significantly, as Case #2 was already in the mild dementia stage, leading to more pronounced deficits. Case #3, presented with mild cognitive impairment (MCI), with normal cognitive screening results but impaired verbal initiative and episodic memory on comprehensive neuropsychological assessment, alongside behavioral and personality changes meeting criteria for mild behavioral impairment (MBI). Concordantly, this patient showed a normal cerebrospinal fluid biomarker profile, including NfL levels, which are expected to become elevated only after the onset of FTD symptoms. Case #4, showed a phenotype compatible with a Lewy body dementia with impairments in visuoconstructive abilities and episodic memory detected on MMSE screening. Interestingly, asymptomatic Case #6 is the offspring of Case #4 and shares the same intermediate allele size as his father and no cognitive assessment was performed. Unfortunately, CSF was not available for biomarker analysis in any of these two individuals.

**Table 1:** Demographic characteristics of the population.

	FTD/ALS spectrum	Asymptomatic	*p*-value
*n*	610	63	–
Gender F/M	269/341	38/25	0.014
Mean Age ± SD, years	62 ± 12	40 ± 15	< 0.001
Clinical subtypes			
FTD	373	–	–
FTD-ALS	34	–	–
ALS	112	–	–
FTD-atypical parkinsonian features	38	–	–
AD	53	–	–
C9RE+	65	24	–

F, Female; M, Male; SD, standard deviation; FTD, Frontotemporal Dementia; FTD-ALS, Frontotemporal Dementia with Amyotrophic Lateral Sclerosis; ALS, Amyotrophic Lateral Sclerosis; AD, Alzheimer’s Disease; C9RE, *C9orf72* repeat expansion.

**Table 2:** Clinical, neuropsychological, and biomarker characteristics of seven subjects carrying intermediate *C9orf72* alleles.

Case	Gender	Age	Diagnosis	Alleles length	Neuropsychological evaluation		NfL evaluation		CSF-AD biomarkers evaluation				
					MMSE Score	MoCA score	CSF NfL (pg/mL)	NfL-category	CSF Ab42 (pg/mL)	CSF Ab42/40	CSF Tau (pg/mL)	CSF pTau181 (pg/mL)	ATN profile
#1	M	55	FTD-ALS	7, 21	—	—	5,507	High	642	0,088	101	21,8	A – T – N −
#2	M	65	PPA and language-variant AD	2, 23	12	7	1,129	High	646	0,052	862	152,6	A + T + N+
#3	F	56	MCI-MBI	8, 22	29	25	307	Normal	1,254	0,110	219	31,9	A-T-N-
#4	M	82	Lewy body dementia	2, 20	26	—		—	—	—	—	—	—
#5	F	70	FTD-ALS	8, 22	11	—	5,522	High	950	0,104	227	25,4	A − T − N −
#6	F	48	Asymptomatic	7, 20	—	—	—	—	—	—	—	—	—
#7	M	63	PPA and language-variant AD	7, 20	27	23	2054	High	881	0,059	468	76,9	A + T + N+

M, Male; F, Female; FTD-ALS, Frontotemporal dementia with amyotrophic lateral sclerosis; PPA, Primary progressive aphasia; MCI, Mild Cognitive Impairment; MBI, Mild Behavioral Impairment; MMSE, Mini-Mental State Examination; MoCA, Montreal Cognitive Assessment; NfL, Neurofilament light chain; CSF, Cerebrospinal fluid; ATN, Amyloid, Tau, Neurodegeneration. Biomarker values are dichotomized as abnormal (+) or normal (−).

## 4 Discussion

In this study, we investigated *C9orf72* repeat expansions in 673 individuals, including 610 patients within the FTD/ALS spectrum and 63 asymptomatic relatives, representing one of the largest Portuguese diagnostic cohorts analyzed over a 10-year period. Pathogenic *C9orf72* repeat expansions were identified in 65 patients (11%), with the highest frequencies observed in FTD-ALS (21%), followed by ALS (14%) and FTD (11%). These findings are consistent with previous Western European studies (van der Zee et al., 2013) and further support *C9orf72* as the most frequent genetic cause of FTD and ALS in Portugal. In agreement with previous Portuguese studies (Gromicho et al., 2018; Moore et al., 2020), our results reinforce the importance of routine *C9orf72* genetic testing in patients with FTD, ALS, or combined FTD-ALS phenotypes, in accordance with current recommendations (Roggenbuck, 2020). Conversely, the absence of *C9orf72* expansions in patients with PSP, CBD, or AD suggests that these disorders have a distinct genetic basis in the Portuguese population, consistent with previous reports (Ticozzi et al., 2014).

The identification of *C9orf72* expansion carriers also has important implications for genetic counseling. Under Portuguese guidelines and national legislation, predictive testing may be offered to at-risk adult relatives within a multidisciplinary genetic counseling framework (Paneque et al., 2019). This is particularly relevant given the considerable phenotypic heterogeneity and age-dependent penetrance associated with *C9orf72* expansions. Although penetrance was initially considered almost complete by 80 years of age, more recent studies indicate that it is incomplete, highlighting the need for individualized risk assessment during genetic counseling (Murphy et al., 2017; Crook et al., 2019).

An important contribution of this study is the characterization of *C9orf72* repeat-length distribution in a Portuguese cohort. Although the precise cutoff distinguishing normal from pathogenic alleles remains controversial, most laboratories currently classify alleles containing <20 repeats as normal, 20–30 repeats as intermediate, and ≥30 repeats as pathogenic. The most frequent alleles in our cohort contained 2, 5, and 8 repeat units, closely resembling the distribution reported in other European populations (van der Zee et al., 2013; Iacoangeli et al., 2019; Giardina et al., 2024). Likewise, alleles containing 14–18 repeats were uncommon, whereas alleles with 3 or 19 repeats were not detected, consistent with observations in the Flanders-Belgian population (van der Zee et al., 2013). These findings suggest that the distribution of normal *C9orf72* alleles in the Portuguese population is broadly comparable to that reported elsewhere in Europe. Unfortunately, the absence of a healthy control cohort analyzed using the same methodology precluded direct comparison of *C9orf72* allele size distributions between patients and controls.

Methodologically, the combination of STR-PCR, repeat-primed PCR, and commercial fragment-length analysis enabled reliable identification of pathogenic *C9orf72* expansions in our laboratory. Although this approach accurately detected expansion carriers, it did not allow precise sizing of alleles larger than 145 repeats. Given the complete concordance observed among both assays (RP-PCR and kit-based assays) in detecting expanded alleles, our findings support the robustness of the diagnostic workflow. Accordingly, future diagnostic testing will rely only on the commercial assay following initial STR-PCR screening, facilitating harmonization and the implementation of a standardized protocol across laboratories. In our patient’s cohort, only three patients carried short expansions comprising 38, 63, and 78 repeats, whereas all other expansion carriers harbored alleles larger than 145 repeats.

Notably, there appears to be a gap between shorter pathogenic repeat sizes and large expansions, likely reflecting the high dynamic instability of these alleles, which tend to either expand or contract (Thys and Wang, 2015; Gijselinck et al., 2016; Van Mossevelde et al., 2017; Jackson et al., 2020). Indeed, this instability may occur both between generations (intergenerational instability) and across tissues within the same individual (somatic instability). In fact, repeat size has been reported to vary markedly between tissues, often being larger in central nervous system (CNS) tissue than in blood-derived DNA, on which most studies are based, and can even differ across brain regions within the same individual (van Blitterswijk et al., 2013; Nordin et al., 2015). The clinical significance of this variability remains unclear, raising important questions about the most appropriate testing strategies to ensure accurate interpretation of results in clinical practice. Recently, targeted long-read sequencing identified somatic *C9orf72* repeat expansions arising from wild-type, intermediate, and short pathogenic alleles in ALS and FTD cases (Zhou et al., 2026), suggesting that somatic expansion may contribute to sALS and sFTD. Whether this process exhibits cell-type specificity, similar to that described for *HTT* repeat expansion, remains to be established (Handsaker et al., 2025).

In addition, we also identified intermediate alleles containing 20–23 repeat units in six patients with heterogeneous clinical presentations and in one asymptomatic relative. The clinical significance of these alleles remains uncertain and is still actively debated, as no universally accepted threshold distinguishes normal from pathogenic *C9orf72* repeat lengths. Nevertheless, several studies have reported segregation of 20–29 repeat alleles with FTD and ALS, with affected individuals showing clinical phenotypes similar to those carrying >30 repeats. Together with functional evidence demonstrating progressive reductions in *C9orf72* transcription and increased promoter methylation as repeat length increases, reaching a maximal 52% reduction in transcriptional activity for alleles carrying 24 repeats compared with the 2-repeat wild-type allele (Gómez-Tortosa et al., 2013; van der Zee et al., 2013; Gijselinck et al., 2016). Since then, additional studies in ALS have also reported a significant association between intermediate alleles and disease risk and a meta-analysis of previously published data has further supported this relationship (Iacoangeli et al., 2019). In the present study, comprehensive clinical, neuropsychological, and biomarker evaluations of these seven carriers of intermediate alleles suggest that alleles within this repeat range may contribute to disease susceptibility across a broad spectrum of neurodegenerative phenotypes. However, these findings require confirmation in case–control studies before firm conclusions can be drawn. Furthermore, we found no evidence of intergenerational instability of intermediate alleles in one family, in which both the father (Case #4) and son (Case #6) carried the same 20-repeat allele. This observation is consistent with previous reports suggesting that intermediate alleles may remain stable across generations (van der Zee et al., 2013).

### 4.1 Study limitations

This study has several limitations. First, although it reflects the spectrum of *C9orf72* repeat lengths in our cohort of patients with clinical presentations within the FTD/ALS spectrum, an appropriately matched healthy control group or a well-characterized reference population analyzed using the same methodology was not available, precluding direct comparisons of allele frequencies between patients and the general population. Consequently, larger studies including a well-characterized control population will be required to determine whether alleles containing 20–23 repeats are enriched among patients and to clarify their potential pathogenic significance. Second, although the molecular methods used reliably detected pathogenic expansions, they did not permit accurate sizing of very large expansions (>145 repeats) or sequence-level characterization of repeat interruptions, which may influence repeat stability, amplification efficiency, and clinical penetrance.

## 5 Conclusion

This study demonstrates that *C9orf72* hexanucleotide repeat expansions are an important genetic cause of FTD, ALS, and FTD-ALS in central Portugal and highlights the value of routine genetic testing for accurate diagnosis, genetic counseling, and clinical management. The distribution of repeat lengths in this Portuguese cohort is broadly consistent with other European populations, whereas the absence of expansions in PSP, CBD, and AD supports a distinct genetic basis for these disorders. Finally, our findings suggest that alleles containing 20–23 repeats may contribute to disease susceptibility across a broad spectrum of neurodegenerative phenotypes. However, future multicenter longitudinal studies integrating genetic, clinical, and biomarker data will be essential to validate these observations, establish the pathogenic and prognostic relevance of intermediate repeat alleles, and clarify their role in neurodegenerative disease susceptibility.

## Data Availability

The raw data supporting the conclusions of this article will be made available by the authors, without undue reservation.
